# The expression of cytoglobin as a prognostic factor in gliomas: a retrospective analysis of 88 patients

**DOI:** 10.1186/1471-2407-13-247

**Published:** 2013-05-20

**Authors:** Hong-Wu Xu, Yue-Jun Huang, Ze-Yu Xie, Lan Lin, Yan-Chun Guo, Ze-Rui Zhuang, Xin-Peng Lin, Wen Zhou, Mu Li, Hai-Hua Huang, Xiao-Long Wei, Kwan Man, Guo-Jun Zhang

**Affiliations:** 1 Department of Neurosurgery, Second Affiliated Hospital of Shantou University Medical College, North Dongxia Rd, Shantou, Guangdong, 515041, China; 2 Research Center for Translational Medicine, Second Affiliated Hospital of Shantou University Medical College, North Dongxia Rd, Shantou, Guangdong, 515041, China; 3 Department of pediatrics, Second Affiliated Hospital of Shantou University Medical College, North Dongxia Rd, Shantou, Guangdong, 515041, China; 4 Department of pathology, Second Affiliated Hospital of Shantou University Medical College, North Dongxia Rd, Shantou, Guangdong, 515041, China; 5 Department of pathology, Cancer Hospital of Shantou University Medical College, Raoping Rd, Shantou, Guangdong, 515031, China; 6 Department of Surgery and Centre for Cancer Research, LKS Faculty of Medicine, The University of Hong Kong, Pokfulam, Hong Kong, China; 7 The Breast Center, Cancer Hospital of Shantou University Medical College, Raoping Rd, Shantou, Guangdong, 515031, China

**Keywords:** Glioma, Cytoglobin, Phosphatidylinositol-3 kinase, Recurrence, Prognosis

## Abstract

**Background:**

Evidence suggests that cytoglobin (Cygb) may function as a tumor suppressor gene.

**Methods:**

We immunohistochemically evaluated the expression of Cygb, phosphatidylinositol-3 kinase (PI-3K), phosphorylated (p)-Akt, Interleukin-6 (IL-6), tumor necrosis factor-α (TNFα) and vascular endothelial growth factor (VEGF) in 88 patients with 41 high-grade gliomas and 47 low-grade gliomas. Intratumoral microvessel density (IMD) was also determined and associated with clinicopathological factors.

**Results:**

Low expression of Cygb was significantly associated with the higher histological grading and tumor recurrence. A significant negative correlation emerged between Cygb expression and PI3K, p-Akt, IL-6, TNFα or VEGF expression. Cygb expression was negatively correlated with IMD. There was a positive correlation between PI3K, p-Akt, IL-6, TNFα and VEGF expression with IMD.High histologic grade, tumor recurrence, decreased Cygb expression, increased PI3K expression, increased p-Akt expression and increased VEGF expression correlated with patients’ overall survival in univariate analysis. However, only histological grading and Cygb expression exhibited a relationship with survival of patients as independent prognostic factors of glioma by multivariate analysis.

**Conclusions:**

Cygb loss may contribute to tumor recurrence and a worse prognosis in gliomas. Cygb may serve as an independent predictive factor for prognosis of glioma patients.

## Background

Glioma is the most common brain tumor in adults. Its ability to evade immune surveillance and impede anti-tumor responses leads to sustained growth and enhanced malignancy [1,2]. Increasing evidence indicates that cytoglobin (Cygb) and the cytokine influences several aspects of gliomas [3-5]. Cygb is the fourth member of the vertebrate globin family and was identified independently by three groups shortly thereafter [6]. The functions of Cygb remain to be elucidated; however, it may include detoxification of reactive oxygen species (ROS) and scavenging NO [7,8]. Although the function of Cygb in vivo remains largely unknown, decreased expression of Cygb and the hypermethylation of the Cygb promoter has been reported in patients with tylosis, non–small-cell lung carcinomas, head and neck cancers, ovarian cancers, and breast cancers [9-12]. Those results suggest that Cygb may function as a tumor suppressor gene [13].

Cygb loss has been reported to be associated with increased cancer cell proliferation, elevated extracellular signal–regulated kinase and Akt activation, overexpression of interleukin-6 (IL-6) [14]. Deregulated signaling through phosphatidylinositol-3 kinase (PI-3K)/Akt pathways has been implicated in the malignant transformation of glial cells [15]. Akt is known to regulate actin cytoskeleton reorganization that plays role in tumor cell migration and invasion [16], and inhibition of Akt prevents glioma cell growth [17]. IL-6 is implicated as major regulators of glioma cell growth and invasiveness. IL-6 regulates the immune response, preferentially activates the signal transducer and activator of transcription-3 (STAT-3), leading to dimerization, nuclear translocation and binding to IFN-c-activated site-like DNA elements [18]. IL-6 cytokine has been extensively studied in astroglial tumors at the mRNA [19] and protein level [20,21] and has been proposed as a determinant of brain tumor progression [22]. It has been shown in experimental models that development of glioblastoma requires the presence of IL-6 [23]. Knowing that IL-6 functions as a downstream mediator for tumor necrosis factor-α (TNF-α) [24], IL-6 is also recognized as potent regulators of angiogenesis [vascular endothelial growth factor (VEGF)] [25]. However, in gliomas, few previous studies have focused on the correlation between Cygb and VEGF. The relationship between Cygb and production of immunosuppressive cytokines (IL-6, TNFα, et al) by tumor cells in gliomas also needs to be confirmed. The paucity of prognostic information regarding Cygb expression and the prognostic role of PI3K/Akt signaling in gliomas prompted us to undertake the present study.

In the clinical setting, the histological grading is a key factor for predicting the biological behavior of gliomas and influencing the choice of therapies, particularly determining the use of adjuvant radiation and specific chemotherapy protocols [26,27]. However, to our knowledge, the exact relationship between histological grading and Cygb expression in tumor cells of glioma has not yet been elucidated. In addition, histological grading makes a contribution toward an estimate of recurrence in gliomas, while a possible relationship between Cygb and glioma recurrence remains to be confirmed.

Therefore, the first goal of this study was to determine Cygb, PI3K, phosphorylated (p)-Akt, IL-6, TNFα and VEGF expression in gliomas. And the second goal of this study was to examine the interaction between Cygb- PI3K/Akt signaling and cytokines (IL-6, TNFα, VEGF), to assess possible relationships of these molecules with clinicopathological features and patients’ survival.

## Methods

### Patient’s description

The study was carried out in patients with histologically confirmed high and low grade gliomas operated on in the Department of Neurosurgery of Second Affiliated Hospital of Shantou University Medical College between January of 2002 and December of 2011. 88 patients were selected according to our inclusion criteria, which were as follows: All patients with intracranial gliomas for whom archival primary tumor material at diagnosis, age over 18 years old, no previous history of any tumor, no administration of antiepileptic drugs or steroids for more than 3 days, no chemotherapy or radiotherapy received before surgery. Histological sections of the resected primary specimens were reviewed by a senior pathologist according to the criteria of WHO histological classification [28]. All the enrolled patients had received brain tumor resection. After surgery, the patients with glioblastoma multiforme and anaplastic astrocytoma (WHO grade IV and III) were treated with teniposide (70 mg/m^2^ /day, 3 consecutive days during each 42-day cycle) for 4-6 cycles. The patients with astrocytoma (WHO grade II) were treated with teniposide for 1 cycle only after the initial surgery. The patients with oligodendrogliomas and oligoastrocytoma underwent PCV chemotherapy [procarbazine, methyl-1-(2-chloroethyl)-1-nitrosourea (CCNU), and vincristine] every 6 weeks (42-day cycles) for 2-5 cycles [29]. In the study, written informed consents were obtained for all patients, and the study was approved by the Medical Ethics Committee of the Second Affiliated Hospital Shantou University Medical College.

### Immunohistochemical staining and scoring

Three continual sections of 4-5μm sections were subjected to immunostaining using a SP Kit (DAKO, Denmark). Slides were deparaffinized in xylene and rehydrated in decreasing concentrations of ethanol and rinsed in phosphate-buffered saline. The slides were incubated with hydrogen peroxide for 20 min following microwave heating with 10 mM citrate buffer (pH 6.0; Sigma-Aldrich, Germany) at 2-min intervals for a total of 10 min. After blocking with normal serum for 30 min, the slides were incubated with rabbit polyclonal antibody. The following antibodies were used: anti-Cygb diluted 1:300(bs-0590R, Bioss), anti-PI3K diluted 1:200(bs-0128R, Bioss), anti-Akt diluted 1:200(bs-0115R, Bioss), anti-IL-6 diluted 1:300(bs-0781R, Bioss), anti-TNFα diluted 1:300(bs-0078R, Bioss), and anti-VEGF diluted 1:200(RAB-0157, Maixin_Bio). In addition, all cases had been stained CD34 for microvessel counting using anti-CD34 antibody diluted 1:100(bs-2038R, Bioss). All rabbit polyclonal antibodies used were provided by Biosynthesis (Beijing, China). The incubation time was 1 h at room temperature for CD34 and 20 h at 4°C for Cygb, PI3K, p-Akt, IL-6, TNFα and VEGF. Slides were detected by SP Kit for 30 min at room temperature and followed by developing with diaminobenzidine for visualization. Negative controls included sections where primary antibody had been substituted by nonimmune serum.

Cygb, PI3K, p-Akt, IL-6, TNFα and VEGF immunoreactivity was evaluated by light microscopy by two experienced pathologists without knowledge of the clinical information. If a discrepancy occurred between the assessments of the two observers, the slides were reassessed in a combined session without information of the previous scores. In each section, the percentage of tumor cells with Cygb, PI3K, p-Akt, IL-6, TNFα and VEGF immunoreactivity was calculated in at least 500 cells counted in several randomly chosen high power fields. In each case, the percentage of tumor cells with Cygb, PI3K, p-Akt, IL-6, TNFα and VEGF immunoreactivity was the mean value of the 3 continual sections. High expression of proteins was more than median value of tumor cells with positive staining, whereas low expression was less than median value. Intratumoral microvessel density (IMD) was observed in areas of most intense neovascularization or hotspots in the tumor by light microscopy. After the area of the highest neovascularization was determined, single microvessels were manually counted on a × 200 field by two independent observers without knowledge of the patient outcome. Any brown-stained endothelial cell or cell cluster that was clearly separated from adjacent microvessels was considered as a single, countable microvessel, and the IMD value of each sample was the mean of the independent microvessels counts by two observers.

### Statistical analysis

All statistical analysis was carried out by SPSS 17.0 software for Windows. In the basic statistical analysis Cygb, PI3K, p-Akt, IL-6, TNFα and VEGF expressions were treated as continuous variables to avoid any “data-driven” categorization. Associations of Cygb, PI3K, p-Akt, IL-6, TNFα and VEGF expression with clinicopathological characteristics were tested using non-parametric tests with correction for multiple comparisons (Kruskal–Wallis ANOVA, Mann–Whitney U-test and Spearman’s rank correlation coefficient). Correlations among Cygb, PI3K, p-Akt, IL-6, TNFα and VEGF and microvascular parameters were tested with Spearman’s correlation coefficient. Vascular density was given as the mean ± SD as indicated. Data were analyzed by one-way ANOVA with Dunnett’s post hoc test and Turkey’s post hoc test for multigroup comparisons. The survival curve of patients was determined by the Kaplan–Meier method and Cox regression, and statistical evaluation was performed using the log rank test. All results with a two-sided *p* level < 0.05 were considered statistically significant.

## Results

### Patients characteristics

The patients were 51 males and 37 females with median age of 41 years old (range 18–74). The high grade group included 15 patients with glioblastoma multiforme (GBM), (WHO grade IV), 26 patients with anaplastic gliomas (AG), (WHO grade III) and 47 patients with low grade gliomas (LGG), (WHO grade I–II). The followed-up was made by telephone call or clinic with median of 20 months (3-80 months). 43 disease-specific deaths were recorded during follow-up.

### *Immunohistochemical assessment of Cygb, PI3K, p-Akt, IL-6, TNF*α *and VEGF in gliomas*

**Figure 1:**
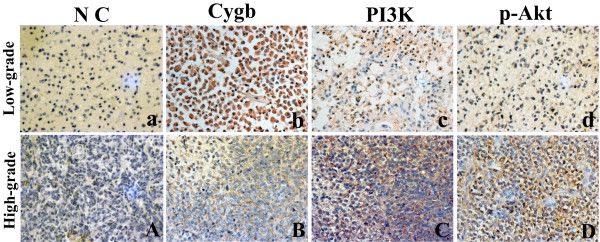
**Immunohistochemical staining of Cygb, PI3K and p-Akt in low and high grade of glioma tissues.** Negative control (NC) of low (**a**) and high (**A**) grade gliomas showed no positive staining cells. Strong and diffused expression of Cygb was found in low-grade gliomas (**b**); in high-grade gliomas, positive staining of Cygb was shown focally and weakly (**B**). Low-grade gliomas showed low expression of PI3K (**c**) and p-Akt (**d**) positive tumor cells in serial section. High expression of PI3K (**C**) and p-Akt (**D**) was shown in high-grade gliomas. (magnification: ×400).

**Figure 2:**
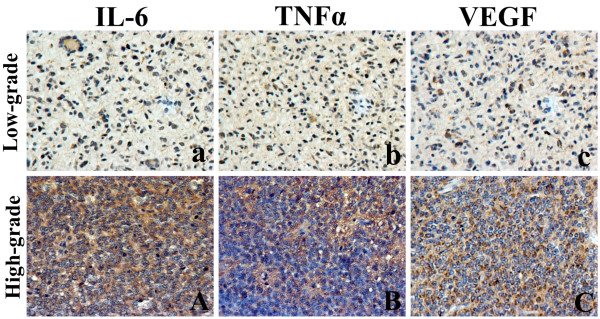
**Immunohistochemical staining of IL-6, TNFα and VEGF in low and high grade of glioma tissues.** Low-grade gliomas showed low expression of IL-6 (**a**), TNFα (**b**) and VEGF (**c**) positive tumor cells in serial section. High expression of IL-6 (**A**), TNFα (**B**) and VEGF (**C**) was shown in high-grade gliomas. (magnification: ×400).

Cytoplasmic and nucleus surface positive staining for Cygb, PI3K and p-Akt was observed in tumor cells of gliomas, no positive staining was shown in tumor cells of negative controls (Figure 1). Immunostaining signal of IL-6, TNFα and VEGF was localized in the cytoplasm of tumor cells (Figure 2) and CD34 was localized in endothelial cells of newly formed vessels. On serial section, CYGB, PI3K, p-Akt, IL-6, TNFα and VEGF could be detected in the same area of tumor cells, at least in a part of them.

Positive staining for Cygb, PI3K, p-Akt, IL-6, TNFα and VEGF were observed in 10%-86% (median value: 39%), 3%-62% (median value: 20%), 5%-67% (median value: 21%), 4%-70% (median value: 35%), 3%-67% (median value: 30%) and 10%-89% (median value: 56%), respectively. Low expression of Cygb was significantly associated with the higher histological grading and tumor recurrence. High expression of PI3K, p-Akt, IL-6, TNFα and VEGF were significantly associated with the higher histological grade, and high expression of PI3K, p-Akt and IL-6 were significantly associated with tumor recurrence. There was no correlation observed between Cygb, PI3K, p-Akt, IL-6, TNFα or VEGF expression and age of patients (Table 1). However, Cygb expression was significantly higher in female patients.

**Table 1:** Correlation among Cygb, VEGF, PI3K, p-Akt, IL-6,TNFα expression and clinicopathological parameters of patients with gliomas

**Vareable**	**Age (n = 88)**	**Gender**		**Histological grade**				**Recurrence**	
		**Male (n = 51)**	**Female (n = 37)**	**Grade I (n = 10)**	**Grade II (n = 37)**	**Grade III (n = 26)**	**Grade IV (n = 15)**	**No (n = 31)**	**Yes (n = 57)**
**Cygb expression**									
Median	39%	30%	52%	56.5%	66%	31.5%	14%	64%	22%
Range	10%-86%	11%-85%	10%-86%	20%-86%	12%-85%	18%-52%	10%-20%	25%-86%	10%-85%
p value	0.439^▴^ (r = -0.083)	0.031^△^		<0.01^♦^				<0.01^△^	
**PI3K expression**									
Median	20%	30%	15%	13%	11%	32.5%	38%	12%	27%
Range	3%-62%	3%-62%	3%-60%	3%-37%	3%-58%	7%-60%	24%-62%	3%-56%	5%-62%
p value	0.236^▴^ (r = 0.128)	0.055^△^		<0.01^♦^				<0.01^△^	
**p-Akt expression**									
Median	21%	27%	17%	15%	14%	31.5%	40%	15%	29%
Range	5%-67%	5%-62%	5%-67%	5%-38%	5%-60%	10%-57%	27%-67%	5%-58%	7%-67%
p value	0.135▴ (r = 0.161)	0.091^△^		<0.01^♦^				<0.01^△^	
**IL-6 expression**									
Median	35%	35%	35%	22.5%	27%	30%	46%	25%	40%
Range	4%-70%	5%-70%	4%-68%	4%-50%	7%-68%	8%-70%	12%-66%	4%-68%	5%-70%
p value	0.859^▴^ (r = 0.019)	0.472^△^		0.019^♦^				0.041^△^	
**TNFα expression**									
Median	30%	27%	33%	16%	22%	33%	47%	21%	35%
Range	3%-67%	3%-67%	4%-67%	4%-46%	3%-62%	3%-67%	10%-62%	3%-62%	3%-67%
p value	0.857^▴^ (r = -0.019)	0.543^△^		0.010^♦^				0.123^△^	
**VEGF expression**									
Median	56%	55%	58%	44%	32%	60.5%	67%	30%	61%
Range	10%-89%	10%-87%	11%-89%	11%-78%	10%-85%	10%-87%	12%-89%	10%-85%	10%-89%
p value	0.178^▴^ (r = 0.145)	0.337^△^		0.027^♦^				0.061^△^	

▴ Results Spearman’s correlation coefficient. △ Results Mann–Whitney U test. ♦ Results Kruskal–Wallis ANOVA.

### Correlations between Cygb, PI3K, p-Akt, IL-6, TNFα,and VEGF immunoreactivity in gliomas

A significant negative correlation emerged between Cygb expression and PI3K or p-Akt expression (r = -0.728, p <0.0001 and r = -0.711, p <0.0001 respectively). High PI3K and p-Akt expression was correlated with high IL-6 (r = 0.302, p = 0.004 and r = 0.328, p = 0.002, respectively) and TNFα expression (r = 0.278, p = 0.009 and r = 0.308, p = 0.004, respectively). IL-6 expression levels were positively associated with TNFα expression (r =0.724, p = <0.0001). There was significant negative correlation observed between Cygb expression and IL-6 or TNFα or VEGF expression (r = -0.370, p <0.0001, r = -0.345, p = 0.001 and r = -0.378, p < 0.0001 respectively). High expression of IL-6 and TNFα exhibited a close correlation with high expression of VEGF in the tumor cells (r = 0.714, p < 0.0001 and r =0.702, p < 0.0001 respectively) (Table 2).

**Table 2:** **Spearman’s correlation coefficient between Cygb, PI3K, p-Akt, IL-6, TNF**α**, VEGF expression and IMD value**

	**Cygb**	**PI3K**	**p-Akt**	**IL-6**	**TNFα**	**VEGF**
**PI3K**						
**r**	-0.728					
**p**	<0.0001					
**p-Akt**						
**r**	-0.711	0.818				
**p**	<0.0001	<0.0001				
**IL-6**						
**r**	-0.370	0.302	0.328			
**p**	<0.0001	0.004	0.002			
**TNFα**						
**r**	-0.345	0.278	0.308	0.724		
**p**	0.001	0.009	0.004	<0.0001		
**VEGF**						
**r**	-0.378	0.395	0.406	0.714	0.702	
**p**	<0.0001	<0.0001	<0.0001	<0.0001	<0.0001	
**IMD**						
**r**	-0.514	0.396	0.426	0.710	0.691	0.605
**p**	<0.0001	<0.0001	<0.0001	<0.0001	<0.0001	<0.0001

### *Correlation of Cygb, PI3K, p-Akt, IL-6, TNF*α *and VEGF expression with IMD in gliomas*

Microvessels in gliomas, specifically stained by anti-CD34 immunostaining, were observed in all specimens, and scored as IMD. The mean IMD value was 30/HPF, but with great individual variation (range 12–56). The correlation between IMD and proteins expression in gliomas is shown in Table 2. Cygb expression was negatively correlated with IMD. There was a positive correlation between PI3K, p-Akt, IL-6, TNFα and VEGF expression with IMD. The IMD was significantly higher in tumors with high expression of PI3K, p-Akt, IL-6, TNFα or VEGF than in tumors with lower protein expression (Table 2).

### *Association of Cygb, PI3K, p-Akt, IL-6, TNF*α *and VEGF expression with survival of patients with gliomas*

The 88 cases were followed up from 3 to 80 months with a mean period of 20 months, and 43 patients (48.9%) had died of their tumor during this period. The mean survival time of patients with high-grade tumors (WHO III–IV) and low-grade tumors (WHO I–II) were 17.3 ± 1.7 and 60.7 ± 4.2 months, respectively; there was a statistically significant difference (p <0.01).

**Figure 3:**
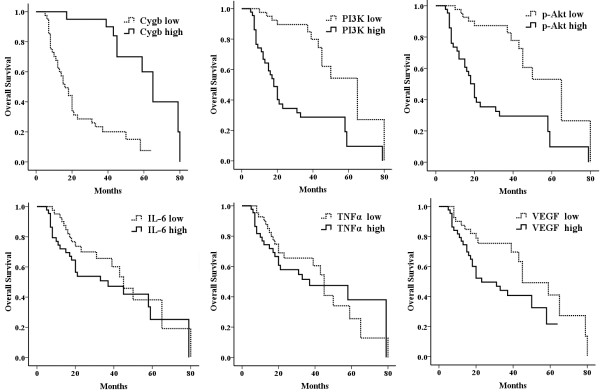
Kaplan-Meier curves estimates of overall survival according to expression of Cygb, PI3K, p-Akt, IL-6, TNFα and VEGF.

Univariate survival analysis (Kaplan–Meier analysis) was carried out in all cases. Figure 3 showed Kaplan–Meier curves of Cygb, PI3K, p-Akt, IL-6, TNFα and VEGF expression with overall survival in the entire case. The parameters adversely affecting survival in all cases were high histologic grade(p < 0.01), tumor recurrence (p < 0.01), decreased Cygb expression(p < 0.01), increased PI3K expression(p < 0.01), increased p-Akt expression (p < 0.01) and increased VEGF expression (p = 0.023) (Table 3). The overall survival is 42.9 ± 3.6 months for all patients. The median survival time was 62.4 ± 4.8 months for patients overexpressing Cygb compared to 23.8 ± 3.1 months for patients with lower expression. The median survival time for patients whose tumors displayed overexpression of PI3K and p-Akt were 29.2 ± 4.2 months and 29.6 ± 4.3 months respectively. The corresponding figure for patients whose tumors presented increased expression of IL-6, TNFα and VEGF were 40.1 ± 5.0 months, 44.2 ± 5.3 and 34.0 ± 3.6 months respectively (Table 3).

**Table 3:** Kaplan–Meier analysis for overall survival rate of patients with gliomas

**Characteristics**	**Mean survival time ± SE**	**95% Confidence interval (months)**	**P values**
**Age (years)**			
**<41**	49.8 ± 5.4	39.2-60.4	0.055
**≥41**	35.7 ± 4.5	26.8-44.5	
**Gender**			
**Male**	39.2 ± 4.6	30.1-48.2	0.086
**Female**	46.2 ±4.3	37.8-54.6	
**Histological grade**			
**Low-grade (WHOI-II)**	60.7 ±4.2	52.5-69.0	<0.01
**High-grade (WHO III-IV)**	17.3 ±1.7	14.0-20.7	
**Tumor recurrence**			
**No**	61.8 ±1.1	59.6-63.9	<0.01
**Yes**	33.1 ± 3.7	25.9-40.3	
**Cygb expression**			
**Low expression (<39%)**	23.8 ±3.1	17.8-29.8	<0.01
**High expression (≥39%)**	62.4 ±4.8	53.0-71.7	
**PI3K expression**			
**Low expression (<20%)**	56.5 ± 5.1	46.4-66.5	<0.01
**High expression (≧%)**	29.2 ± 4.2	20.9-37.5	
**p-Akt expression**			
**Low expression (<21%)**	55.4 ± 5.1	45.3-65.4	<0.01
**High expression (≧20%)**	29.6 ± 4.3	21.2-38.1	
**IL-6 expression**			
**Low expression(<35%)**	45.9 ± 5.2	35.7-56.2	0.157
**High expression (≧35%)**	40.1 ± 5.0	30.3-49.8	
**TNFα expression**			
**Low expression (<30%)**	62.9 ± 4.6	33.8-51.9	0.569
**High expression (≧30%)**	44.2 ± 5.3	33.7-54.6	
**VEGF expression**			
**Low expression (<56%)**	50.1 ± 5.2	39.9-60.4	0.023
**High expression (≧56%)**	34.0 ± 3.6	26.9-41.2	

Multivariate survival analysis (Cox regression model) results including all parameters for the 88 patients, for whom Cygb, PI3K, p-Akt, IL-6, TNFα, VEGF and CD34 staining results were available are presented in Table 4. Only histological grade and Cygb expression appeared to affect survival in all cases (Table 4). Other parameters, such as age, gender, tumor recurrence, angiogenesis and expression of PI3K, p-Akt, IL-6, TNFα and VEGF did not show any association with the survival of the patients (Table 4).

**Table 4:** Cox regression model for multivariate analyses of prognostic factor in gliomas

**Variable**	**Wald**	**Hazard ratio**	**95% Confidence interval**	**P value**
**Age (>41 vs <41)**	0.678	0.722	0.333-1.567	0.410
**Gender (male vs. female)**	0.830	0.696	0.319-1.518	0.362
**Histological grade (low-grade vs. high-grade)**	14.358	15.320	3.734-62.857	<0.01
**Tumor recurrence (no vs. yes)**	3.142	6.383	0.822-49.563	0.076
**Cygb expression (low vs. high)**	5.254	0.235	0.068-0.811	0.022
**PI3K expression (low vs. high)**	0.808	3.161	0.275-38.859	0.369
**p-Akt expression (low vs. high)**	0.323	0.503	0.047-5.374	0.570
**IL-6 expression (low vs. high)**	2.137	2.730	0.710-10.493	0.144
**TNF**α **expression (low vs. high)**	1.818	0.390	0.099-1.532	0.178
**VEGF expression (low vs. high)**	1.119	0.560	0.191-1.641	0.290
**Angiogenesis (IMD value)**	0.375	1.016	0.966-1.068	0.540

## Discussion

Cygb as a tumor suppressor gene has been demonstrated in hepatocellular carcinoma, lung cancer and breast cancer [9-12]. Expression of Cygb has been reported in various human tumors, including gliomas [5]. This study showed that Cygb expression was found to inversely associate with higher histological grade in gliomas, and lower expression of Cygb is closely related to a shorter survival time of patients using either univariate or multivariate analysis. These results indicated that Cygb may function not only as a tumor suppressor gene, which was supported by previous studies, but also as a prognostic factor. Previous studies have indicated that Cygb loss was associated with increased cancer cell proliferation and overexpression of IL-1, IL-6, VEGF, TNFα, and TNFb mRNAs in cancer development in the liver and lungs of mice exposed to N,N-diethylnitrosamine [14]. As we known, IL-1, IL-6, VEGF and TNFα are immunosuppressive cytokines. Immunosuppressive cytokines and tumor biology are closely intertwined since increased production of immunosuppressive cytokines in cancer cells not only had a stimulating effect on tumor cell growth and proliferation but also regarded as an indispensable participation in tumor progression were known to evade immune surveillance [30,31]. We postulated that Cygb was likely to influence the prognosis of glioma patients by effect on production of immunosuppressive cytokines and angiogenesis in gliomas.

In the present study, we found a direct relationship between Cygb expression level and tumor recurrence, independent of vascular density and angiogenic factor expression. In particular, Grade I–II gliomas with lower Cygb expression were found more likely to recur during the period of follow-up despite the low level of proliferative activity in this tumor. In the clinic, neither histopathologic nor clinical data were currently taken as reliable recurrence predictors for low-grade gliomas, especially for grade I tumors. Although the number of glioma samples examined in this study was not sufficient to allow us to draw a definite conclusion, our findings indicated that Cygb expression level could be used as a predictor for the recurrence of gliomas. There are some histological features of gliomas associated with grade and prognosis, such as the extent of vascular density [32]. In our study, we found that low expression of Cygb in glioma cells was closely associated with higher microvessel density in tumor, which suggested Cygb might contribute partly to angiogenesis of gliomas. These results indicated that Cygb loss in tumor cells might play an important role in tumor progression through the induction of angiogenesis. In the present study, we found that IL-6 high expression in gliomas was significantly correlated with low expression of Cygb, as well as higher histological grade and increased neovascularization in tumors.

Previous study found that PI3K/Akt pathway is deregulated in GBM [33] and activation of this pathway has been shown to be associated with reduced patient survival [34]. In our study, we found low level of Cygb expression was associated with an increased Akt and PI3K expression in gliomas. The increased levels of Akt and PI3K correlated with a marked elevation of IL-6 and TNFα. These data suggested that low expression of Cygb, together with high expression of IL-6, TNFα, Akt and PI3K might play an important role in the development of glioma.

In this particular cohort, 62.2% (23 out of 37) of female patients was with low grade tumors while about 47% (24 out of 51) of male patients was diagnosed as low grade tumors. The selection bias was found to cause the gender difference in terms of histological grade. Thus, the finding that low expression of Cygb was significantly associated with female gender was most likely because of difference in histological grade.

In further study, we will investigate the role of Cygb expression by overexpressing via introducing exogenous Cygb or silencing Cygb protein via siRNA in cultured glioma cells. Moreover, we will try to develop animal models of gliomas either by transgenic models or by subcutaneous injection to charcterize the significance of Cygb overexpression or inhibition.

## Conclusion

In conclusion, the present data indicated prognostic significance of Cygb in gliomas: correlation with PI3K, Akt, IL-6, TNFα, VEGF, microvessel morphometry and survival of patients with gliomas. Cygb loss may play an important role in contributing to production of immunosuppressive cytokines and angiogenesis in gliomas. Histological grade and Cygb expression in tumors are independent predictors for the prognosis of patients with gliomas. Moreover, Cygb expression level may help determine whether aggressive therapy is necessary, particularly for those gliomas with lower WHO grades.

## Abbreviations

Cygb: Cytoglobin; PI3K: Phosphatidylinositol-3 kinase; IL-6: Interleukin-6; TNFα: Tumor necrosis factor- α; VEGF: Vascular endothelial growth factor; IMD: Intratumoral microvessel density; ROS: Reactive oxygen species; STAT-3: Signal transducer and activator of transcription-3; DAB: Diaminobenzidine

## Competing interests

The authors declare that they have no competing interests.

## Authors’ contributions

HWX and YJH are co-first authors, and they made equal contributions to this work. HWX performed immunohistochemical staining, data analysis and drafted the manuscript. YJH followed up the cases and carried out the statistical analysis. LL, YCG, ZRZ, XPL, WZ and ML participated in collecting correlative cases. HHH and XLW reviewed the histological sections of the cases. GJZ and ZYX are co-corresponding authors. GJZ conceived of the study and participated in its design. ZYX and KM participated in the overall design. All authors read and approved the final manuscript.

## Pre-publication history

The pre-publication history for this paper can be accessed here:

http://www.biomedcentral.com/1471-2407/13/247/prepub

## References

[B1] Louis DN Molecular pathology of malignant gliomas Annu Rev Pathol 2006 1 97 117 10.1146/annurev.pathol.1.110304.100043 18039109

[B2] Furnari FB Fenton T Bachoo RM Mukasa A Stommel JM Stegh A Hahn WC Ligon KL Louis DN Brennan C Chin L DePinho RA Cavenee WK Malignant astrocytic glioma: genetics, biology, and paths to treatment Genes Dev 2007 21 21 2683 2710 10.1101/gad.1596707 17974913

[B3] Saidi A Hagedorn M Allain N Verpelli C Sala C Bello L Bikfalvi A Javerzat S Combined targeting of interleukin-6 and vascular endothelial growth factor potently inhibits glioma growth and invasiveness Int J Cancer 2009 125 5 1054 1064 10.1002/ijc.24380 19431143

[B4] Sciume G Santoni A Bernardini G Chemokines and glioma: invasion and more J Neuroimmunol 2010 224 1–2 8 12 20656128 10.1016/j.jneuroim.2010.05.019

[B5] Fang J Ma I Allalunis-Turner J Knockdown of cytoglobin expression sensitizes human glioma cells to radiation and oxidative stress Radiat Res 2011 176 2 198 207 10.1667/RR2517.1 21631290

[B6] Burmester T Ebner B Weich B Hankeln T Cytoglobin: a novel globin type ubiquitously expressed in vertebrate tissues Mol Biol Evol 2002 19 4 416 421 10.1093/oxfordjournals.molbev.a004096 11919282

[B7] Fordel E Thijs L Martinet W Schrijvers D Moens L Dewilde S Anoxia or oxygen and glucose deprivation in SH-SY5Y cells: a step closer to the unraveling of neuroglobin and cytoglobin functions Gene 2007 398 1–2 114 122 17532579 10.1016/j.gene.2007.03.022

[B8] Halligan KE Jourd’heuil FL David Jourd’heuil1 Cytoglobin is expressed in the vasculature and regulates cell respiration and proliferation via nitric oxide dioxygenation J Biol Chem 2009 284 13 8539 8547 19147491 10.1074/jbc.M808231200 PMC2659212

[B9] Xinarianos G McRonald FE Risk JM Bowers NL Nikolaidis G Field JK Liloglou T Frequent genetic and epigenetic abnormalities contribute to the deregulation of cytoglobin in non-small cell lung cancer Hum Mol Genet 2006 15 13 2038 2044 10.1093/hmg/ddl128 16698880

[B10] Shaw RJ Hall GL Woolgar JA Lowe D Rogers SN Field JK Liloglou T Risk JM Quantitative methylation analysis of resection margins and lymph nodes in oral squamous cell carcinoma Br J Oral Maxillofac Surg 2007 45 8 617 622 10.1016/j.bjoms.2007.04.015 17559992

[B11] Shaw RJ Omar MM Rokadiya S Kogera FA Lowe D Hall GL Woolgar JA Homer J Liloglou T Field JK Risk JM Cytoglobin is upregulated by tumour hypoxia and silenced by promoter hypermethylation in head and neck cancer Br J Cancer 2009 101 1 139 144 10.1038/sj.bjc.6605121 19568272 PMC2713706

[B12] Gorr TA Wichmann D Pilarsky C Theurillat JP Fabrizius A Laufs T Bauer T Koslowski M Horn S Burmester T Hankeln T Kristiansen G Old proteins – new locations: myoglobin, haemoglobin, neuroglobin and cytoglobin in solid tumours and cancer cells Acta Physiol (Oxf) 2011 202 3 563 581 10.1111/j.1748-1716.2010.02205.x 20958924

[B13] Shivapurkar N Stastny V Okumura N Girard L Xie Y Prinsen C Thunnissen FB Wistuba II Czerniak B Frenkel E Roth JA Liloglou T Xinarianos G Field JK Minna JD Gazdar AF Cytoglobin, the newest member of the globin family, functions as a tumor suppressor gene Cancer Res 2008 68 18 7448 7456 10.1158/0008-5472.CAN-08-0565 18794132 PMC2849650

[B14] le Thuy TT Morita T Yoshida K Wakasa K Iizuka M Ogawa T Mori M Sekiya Y Momen S Motoyama H Ikeda K Yoshizato K Kawada N Promotion of Liver and Lung Tumorigenesis in DEN-Treated Cytoglobin-Deficient Mice Am J Pathol 2011 179 2 1050 1060 10.1016/j.ajpath.2011.05.006 21684245 PMC3157247

[B15] Rao RD James CD Altered molecular pathways in gliomas: an overview of clinically relevant issues Semin Oncol 2004 31 5 595 604 10.1053/j.seminoncol.2004.07.002 15497113

[B16] Qian Y Zhong X Flynn DC Zheng JZ Qiao M Wu C Dedhar S Shi X Jiang BH ILK mediates actin filament rearrangements and cell migration and invasion through PI3K/Akt/Rac1 signaling Oncogene 2005 24 19 3154 3165 10.1038/sj.onc.1208525 15735674

[B17] Gallia GL Tyler BM Hann CL Siu IM Giranda VL Vescovi AL Brem H Riggins GJ Inhibition of Akt inhibits growth of glioblastoma and glioblastoma stem-like cells Mol Cancer Ther 2009 8 2 386 393 10.1158/1535-7163.MCT-08-0680 19208828 PMC4498795

[B18] Shuai K Liu B Regulation of JAK-STAT signaling in the immune system Nat Rev Immunol 2003 3 11 900 911 10.1038/nri1226 14668806

[B19] Tchirkov A Rolhion C Bertrand S Doré JF Dubost JJ Verrelle P IL-6 gene amplification and expression in human glioblastomas Br J Cancer 2001 85 4 518 522 10.1054/bjoc.2001.1942 11506489 PMC2364101

[B20] Samaras V Piperi C Korkolopoulou P Zisakis A Levidou G Themistocleous MS Boviatsis EI Sakas DE Lea RW Kalofoutis A Patsouris E Application of the ELISPOT method for comparative analysis of interleukin (IL)-6 and IL-10 secretion in peripheral blood of patients with astroglial tumours Mol Cell Biochem 2007 304 1–2 343 351 17551671 10.1007/s11010-007-9517-3

[B21] Zisakis A Piperi C Themistocleous MS Korkolopoulou P Boviatsis EI Sakas DE Patsouris E Lea RW Kalofoutis A Comparative analysis of peripheral and localised cytokine secretion in glioblastoma patients Cytokine 2007 39 2 99 105 10.1016/j.cyto.2007.05.012 17697783

[B22] Tchirkov A Khalil T Chautard E Mokhtari K Véronèse L Irthum B Vago P Kémény JL Verrelle P Interleukin-6 gene amplification and shortened survival in glioblastoma patients Br J Cancer 2007 96 3 474 476 10.1038/sj.bjc.6603586 17224923 PMC2360031

[B23] Brantley EC Benveniste EN Signal transducer and activator of transcription-3: a molecular hub for signaling pathways in gliomas Mol Cancer Res 2008 6 5 675 684 10.1158/1541-7786.MCR-07-2180 18505913 PMC3886801

[B24] Kamimura D Ishihara K Hirano T IL-6 signal transduction and its physiological roles: the signal orchestration model Rev Physiol Biochem Pharmacol 2003 149 1 38 12687404 10.1007/s10254-003-0012-2

[B25] Koch AE Polverini PJ Kunkel SL Harlow LA DiPietro LA Elner VM Elner SG Strieter RM Interleukin-8 as a macrophage derived mediator of angiogenesis Science 1992 258 5089 1798 1801 10.1126/science.1281554 1281554

[B26] Nieder C Andratschke N Wiedenmann N Busch R Grosu AL Molls M Radiotherapy for high-grade gliomas. Does altered fractionation improve the outcome? Strahlenther Onkol 2004 180 7 401 407 15241527 10.1007/s00066-004-1220-7

[B27] Nagy M Schulz-Ertner D Bischof M Welzel T Hof H Debus J Combs SE Long-term outcome of postoperative irradiation in patients with newly diagnosed WHO grade III anaplastic gliomas Tumori 2009 95 3 317 324 19688970 10.1177/030089160909500308

[B28] Louis DN Ohgaki H Wiestler OD Cavenee WK Louis DN Astrocytic tumors WHO classification of tumor of the central nervous system 2007 4 Lyon: IARC Press 14 49

[B29] Stupp R Mason WP van den Bent MJ Weller M Fisher B Taphoorn MJ Belanger K Brandes AA Marosi C Bogdahn U Curschmann J Janzer RC Ludwin SK Gorlia T Allgeier A Lacombe D Cairncross JG Eisenhauer E Mirimanoff RO European Organisation for Research and Treatment of Cancer Brain Tumor and Radiotherapy Groups; National Cancer Institute of Canada Clinical Trials Group Radiotherapy plus concomitant and adjuvant Temozolomide for glioblastoma N Engl J Med 2005 352 10 987 996 10.1056/NEJMoa043330 15758009

[B30] Salazar-Onfray F López MN Mendoza-Naranjo A Paradoxical effects of cytokines in tumor immune surveillance and tumor immune escape Cytokine Growth Factor Rev 2007 18 1–2 171 182 17329145 10.1016/j.cytogfr.2007.01.015

[B31] Mumm JB Oft M Cytokine-based transformation of immune surveillance into tumor-promoting inflammation Oncogene 2008 27 45 5913 5919 10.1038/onc.2008.275 18836472

[B32] Barker FG Davis RL Chang SM Prados MD Necrosis as a prognostic factor in glioblastoma multiforme Cancer 1996 77 6 1161 1166 10.1002/(SICI)1097-0142(19960315)77:6<1161::AID-CNCR24>3.0.CO;2-Z 8635139

[B33] Parsons DW Jones S Zhang X Lin JC Leary RJ Angenendt P Mankoo P Carter H Siu IM Gallia GL Olivi A McLendon R Rasheed BA Keir S Nikolskaya T Nikolsky Y Busam DA Tekleab H Diaz LA Jr Hartigan J Smith DR Strausberg RL Marie SK Shinjo SM Yan H Riggins GJ Bigner DD Karchin R Papadopoulos N Parmigiani G Vogelstein B Velculescu VE Kinzler KW An integrated genomic analysis of human glioblastoma multiforme Science 2008 321 5897 1807 1812 10.1126/science.1164382 18772396 PMC2820389

[B34] Pelloski CE Lin E Zhang L Yung WK Colman H Liu JL Woo SY Heimberger AB Suki D Prados M Chang S Barker FG 3rd Fuller GN Aldape KD Prognostic associations of activated mitogen-activated protein kinase and Akt pathways in glioblastoma Clin Cancer Res 2006 12 13 3935 3941 10.1158/1078-0432.CCR-05-2202 16818690

